# Prevalence and Trends of the Abdominal Aortic Aneurysms Epidemic in General Population - A Meta-Analysis

**DOI:** 10.1371/journal.pone.0081260

**Published:** 2013-12-02

**Authors:** Xi Li, Ge Zhao, Jian Zhang, Zhiquan Duan, Shijie Xin

**Affiliations:** 1 Department of Vascular and Thyroid Surgery, the First Affiliated Hospital of China Medical University, Shenyang, China; 2 Department of Obstetrics, Chinese People's Liberation Army 463th Hospital, Shenyang, China; University of Texas Health Science Center at San Antonio, United States of America

## Abstract

**Objective:**

To conduct a meta-analysis assessing the prevalence and trends of the abdominal aortic aneurysms (AAA) epidemic in general population.

**Method:**

Studies that reported prevalence rates of AAA from the general population were identified through MEDLINE, EMBASE, Web of Science, and reference lists for the period between 1988 and 2013. Studies were included if they reported prevalence rates of AAA in general population from the community. In stratified analyses possible sources of bias, including areas difference, age, gender and diameter of aneurysms were examined. Publication bias was assessed with Egger's test method.

**Results:**

56 studies were identified. The overall pooled prevalence of AAA was 4.8% (4.3%, 5.3%). Stratified analyses showed the following results, areas difference: America 2.2% (2.2%, 2.2%), Europe 2.5% (2.4%, 2.5%), Australia 6.7% (6.5%, 7.0%), Asia 0.5% (0.3%, 0.7%); gender difference: male 6.0% (5.3%, 6.7%), female 1.6% (1.2%, 1.9%); age difference: 55–64years 1.3% (1.2%, 1.5%), 65–74 years 2.8% (2.7%, 2.9%), 75–84 years1.2%(1.1%, 1.3%), ≥85years0.6% (0.4%, 0.7%); aortic diameters difference: 30–39 mm, 3.3% (2.8%, 3.9%), 40–49 mm,0.7% (0.4%,1.0%), ≥50 mm, 0.4% (0.3%, 0.5%). The prevalence of AAA has decreased in Europe from 1988 to 2013. Hypertension, smoking, coronary artery disease, dyslipidemia, respiratory disease, cerebrovascular disease, claudication and renal insufficiency were risk factors for AAA in Europe.

**Conclusion:**

AAA is common in general population. The prevalence of AAA is higher in Australia than America and Europe. The pooled prevalence in western countries is higher than the Asia. Future research requires a larger database on the epidemiology of AAA in general population.

## Introduction

Abdominal aortic aneurysm (AAA) is the pathologic local dilation of the abdominal aorta [1] and is defined as an aorta size more than 30 mm or a local dilation of abdominal aorta more than 50%, as compared to another site along the aorta. Epidemiological studies of AAA have shown an increased incidence worldwide, ranging from 4.2% to 11% per year. [2]–[5] Despite the evolution of our understanding and treatment of AAA in the past few decades, it continues to be a major threat to health because of grave outcome with 80% overall mortality in event of rupture. [6] Early identification of patients with AAA and offer of timely elective repair remain to be the most reliable strategy for prevention of death from ruptured AAA. The benefit of screening for AAA in elderly men had been proven by large-scale randomized studies that reported 50% reduction of AAA rupture and associated mortality. [7]–[9] However, specific information on the prevalence of AAA that would be useful for health services planning has been difficult to establish. To date, the epidemiological studies published have adopted different methodologies for case ascertainment and have demonstrated widely different prevalence that has varied between regions. Whether this variance reflects differences in biological substrates or the methodological approaches of each study has been difficult to determine. Thus far, no meta-analysis on the prevalence or trends of abdominal aortic aneurysms in general population exists. Accordingly, the aim of this study was to assess the prevalence rates of abdominal aortic aneurysms in the general population and to describe the secular trends in this prevalence from 1988 to 2013, and to examine potential moderator variables that may impact heterogeneity in prevalence rates.

## Methods

This meta-analysis included cross-sectional studies, randomized controlled trials and prospective cohort studies which reported data involving the prevalence of patients with AAA. This study was conducted in accordance with the ‘preferred reporting items for systematic reviews and meta-analyses’ (Checklist S1) guidelines. No protocol exists for this meta-analysis.

### Search Strategy

We assessed all English and Chinese publications that reported the prevalence of AAA among worldwide populations. We searched the electronic databases of MEDLINE, EMBASE, Web of Science for relevant papers published from 1988 through 2013. The search keywords were: abdominal aortic aneurysm, prevalence. A manual search was performed by checking the reference lists of original reports and review articles, retrieved through the electronic searches, to identify studies not yet included in the computerized databases.

### Inclusion and Exclusion Criteria

The inclusion criteria were:

1. Studies in the mentioned three databases with full text;
2. Population-based studies;
3. Studies provided sufficient information to estimate the pooled prevalence of AAA.

The exclusion criteria were:

1. Studies without specific sample origins;
2. Studies with overlapping time intervals of sample collection from the same origin;
3. Studies with a sample size less than 50;
4. Studies that failed to present data clearly enough or with obviously paradoxical data.

### Data Extraction

All the potentially relevant papers were reviewed independently by two investigators through assessing the eligibility of each article and abstracting data with standardized data-abstraction forms. Disagreements were resolved through discussion. The following information, though some studies did not contain all of them, were extracted from the literatures: first author's name, publication date, country, design, age, gender, number invited, number screened, definition of AAA, risk factors and prevalence rate by different stratified factors, including areas difference, age, gender and diameter of aneurysms.

### Data Analysis

The primary outcome of this meta-analysis was the prevalence rate of AAA, defined as the number of cases divided by the total number of study participants. To examine possible sources of bias, stratified analyses were conducted for the studies. We investigated the effect of potentially distorting factors, including areas difference, age, gender and diameter of aneurysms of included participants. Because of insufficient numbers of studies for individual years, studies were grouped into eight 3-year periods, 1988–1992, 1993–1995, 1996–1998, 1999–2001, 2002–2004, 2005–2007, 2008–2010 and 2011–2013. Publication bias was assessed for the included studies, by visually inspecting funnel plots and applying Egger's test. [10], [11] Risk factor associations were expressed as odds ratios (ORs) to obtain consistency across studies. All analyses were conducted using STATA 12.0 (STATA Corporation, College Station, TX, USA). A random-effects model was chosen for data analysis as this model better addresses heterogeneity between studies and study populations and was less influenced by extreme variations in sample size. Heterogeneity among study prevalence estimates was assessed by means of the *Q* statistic, with magnitude of heterogeneity evaluated with the *I* ^2^ index.

## Results

**Figure 1:**
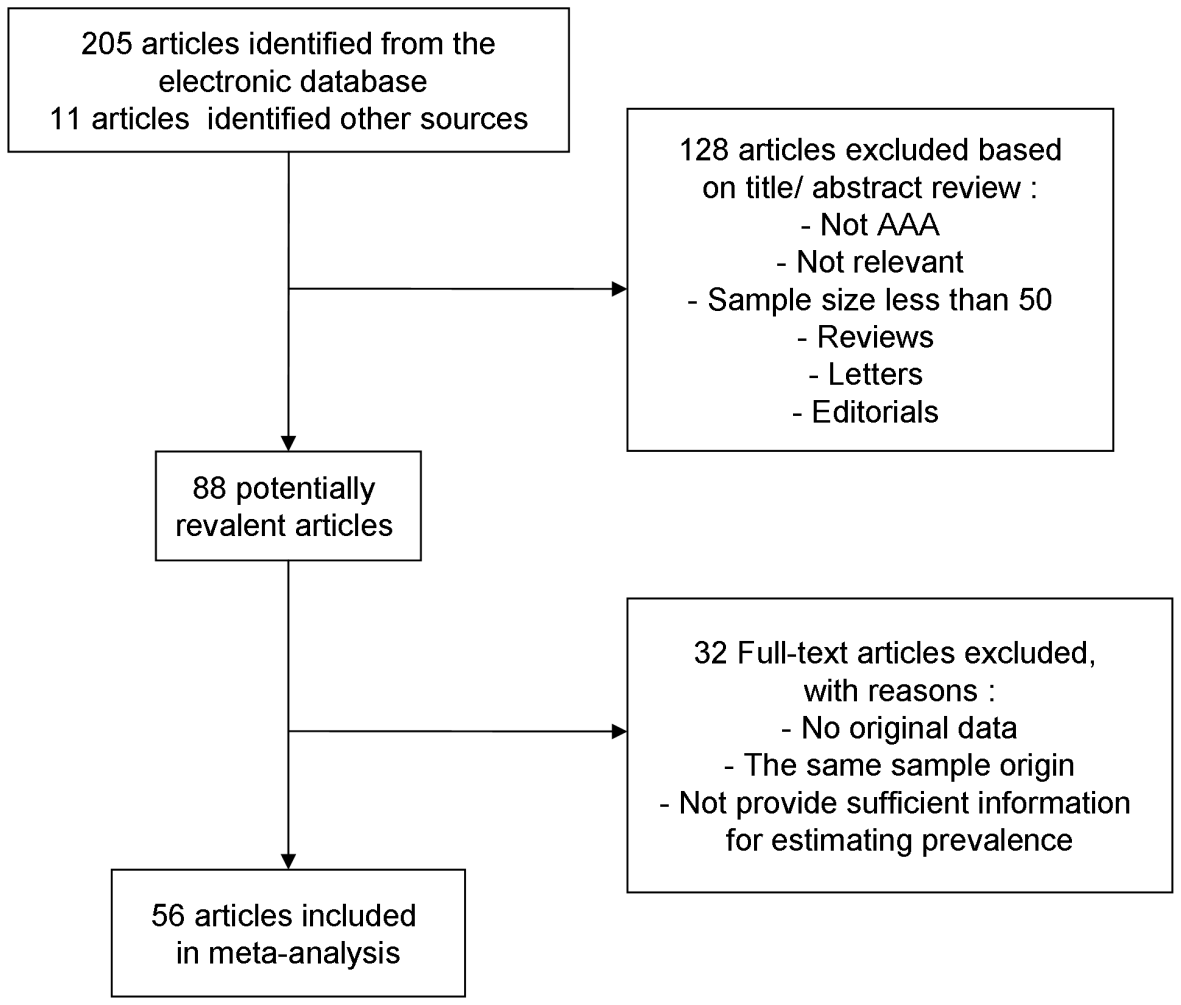
Flow chart demonstrating those studies that were processed for inclusion in the meta-analysis.

**Figure 2:**
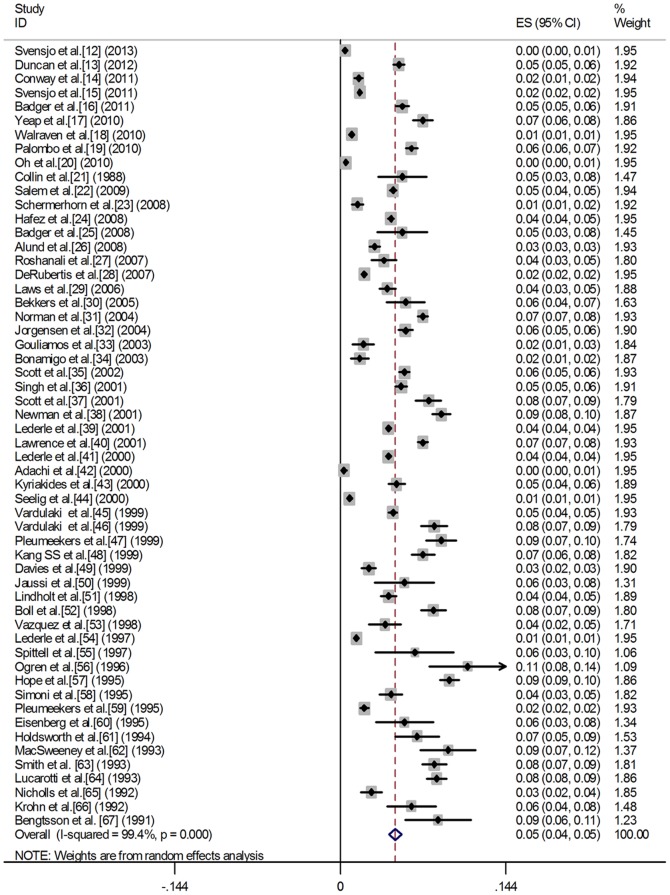
A forest plot of prevalence from individual studies and combined prevalence from random-effects models.

We screened 216 abstracts published from 1988 to 2013 and reviewed a total of 88 full-text articles. Of these, 32 were excluded for the following reasons: no original data, the same sample origin and not provide sufficient information for estimating prevalence. Thus, 56 studies were included in this meta-analysis. Figure 1 gives a schematic representation of the selection process and reasons for excluding studies. The characteristics of the 56 included studies are summarized in table 1. All studies were based on general population samples and used abdominal ultrasound as screening test. Figure 2 shows a forest plot of prevalence from individual studies and combined prevalence from random-effects models. The definitions used for AAA varied between studies, but most studies used a similar definition (a diameter greater than 30 mm).

**Table 1:** Characteristics of population-based studies of abdominal aortic aneurysm.

Serial number	Study	Publication year	Country	Design	Age(years)	Gender	Number invited	Number screened (%)
1	Svensjö et al.[12]	2013	Sweden	CSS	70	W	6925	5140(74.2)
2	Duncan et al.[13]	2012	UK	PCS	65–74	M	8355	8146 (97.5)
3	Conway et al.[14]	2011	UK	CSS	65	M	6091	4216 (69.2)
4	Svensjö et al.[15]	2011	Sweden	CSS	65	M	26256	22139 (84.3)
5	Badger et al.[16]	2011	UK	CSS	65–75	M	13316	5931 (44.5)
6	Yeap et al.[17]	2010	Australia	CSS	70–88	M	n.a.	3620
7	Walraven et al.[18]	2010	Canada	CSS	65–80	M and W	311066	79121 (25)
8	Palombo et al.[19]	2010	Italy	CSS	65–92	M and W	15151	8234 (54.3)
9	Oh et al.[20]	2010	Korea	CSS	12–98	M and W	6267	4939 (79)
10	Collin et al.[21]	1988	UK	CSS	65–74	M	824	426 (51.7)
11	Salem et al.[22]	2009	UK	CSS	65	M	n.a.	19014
12	Schermerhorn et al.[23]	2008	USA	CSS	≥65	M and W	30000	2005 (6.7)
13	Hafez et al.[24]	2008	UK	CSS	64–81	M	350000	22961 (6.6)
14	Badger et al.[25]	2008	UK	CSS	65–75	M	908	409 (45.0)
15	Alund et al.[26]	2008	Sweden	CSS	20–98	M and W	9296	5924 (63.7)
16	Roshanali et al.[27]	2007	Iran	PCS	13–80	M and W	1285	1175 (91.4)
17	DeRubertis et al.[28]	2007	USA	CSS	≥50	M and W	n.a.	17540
18	Laws et al.[29]	2006	UK	CSS	65–80	M	4000	2870(71.7)
19	Bekkers et al.[30]	2005	Netherlands	CSS	Mean = 60.5	M and W	796	742 (93.2)
20	Norman et al.[31]	2004	Australia	RCT	65–83	M	17516	12203 (70)
21	Jørgensen et al.[32]	2004	Norway	CSS	55–74	M and W	5465	5392 (98.7)
22	Gouliamos et al.[33]	2003	Greece	CSS	55–85	M and W	n.a.	850
23	Bonamigo et al.[34]	2003	Brazil	CSS	≥54	M	n.a.	1012
24	Scott et al.[35]	2002	UK	RCT	65–80	M and W	n.a.	9485
25	Singh et al.[36]	2001	Norway	PCS	55–74	M and W	6892	6386 (92.7)
26	Scott et al. [37]	2001	UK	RCT	64–81	M	6058	2212 (36.5)
27	Newman et al.[38]	2001	USA	PCS	≥65	M and W	5888	4734 (80.4)
28	Lederle et al.[39]	2001	USA	CSS	50–79	M and W	n.a.	125722
29	Lawrence et al.[40]	2001	Australia	RCT	26–69	M	19583	12203 (62.3)
30	Lederle et al.[41]	2000	USA	CSS	50–79	M and W	n.a.	52745
31	Adachi et al.[42]	2000	Japan	CSS	35–82	M and W	1881	1591 (84.6)
32	Kyriakides et al.[43]	2000	UK	CSS	65	M	4823	3497 (72.5)
33	Seelig et al.[44]	2000	Germany	CSS	≥50	M and W	14876	13166 (88.5)
34	Vardulaki et al.[45]	1999	UK	RCT	≥50	M	n.a.	11291
35	Vardulaki et al.[46]	1999	UK	RCT	≥65	M and W	5000	2215 (44.3)
36	Pleumeekers et al.[47]	1999	Netherlands	CSS	≥55	M	2217	1771 (79.9)
37	Kang et al.[48]	1999	USA	CSS	Mean = 67	M and W	n.a.	2477
38	Davies et al.[49]	1999	UK	CSS	≥50	M and W	n.a.	2281
39	Jaussi et al.[50]	1999	Lausanne	CSS	Mean = 59	M and W	n.a.	301
40	Lindholt et al.[51]	1998	Denmark	RCT	65–73	M	4404	3342 (75.9)
41	Boll et al.[52]	1998	Netherlands	CSS	60–80	M	2914	2419 (83.0)
42	Vazquez et al.[53]	1998	Belgium	CSS	65 and 75	M	1773	727 (41)
43	Lederle et al.[54]	1997	USA	CSS	50–79	M and W	n.a.	73451
44	Spittell et al.[55]	1997	USA	CSS	≥50	M and W	n.a.	200
45	Ogren et al.[56]	1996	Sweden	CSS	Mean = 74	M	423	343 (81.1)
46	Hope et al.[57]	1995	USA	CSS	65–90	M and W	n.a.	4741
47	Simoni et al.[58]	1995	Italy	CSS	65–75	M and W	2734	1601 (58.5)
48	Pleumeekers et al.[59]	1995	Netherlands	CSS	≥55	M and W	10215	5419 (53)
49	Eisenberg et al.[60]	1995	USA	CSS	13–94	M and W	n.a.	323
50	Holdsworth et al.[61]	1994	UK	CSS	65–79	M	800	628 (78.5)
51	MacSweeney et al.[62]	1993	UK	CSS	n.a.	M and W	n.a.	561
52	Smith et al.[63]	1993	UK	CSS	65–75	M	3500	2669 (76)
53	Lucarotti et al.[64]	1993	UK	CSS	65	M	5337	4232 (79)
54	Nicholls et al.[65]	1992	Australia	CSS	60–80	M and W	n.a.	1225
55	Krohn et al.[66]	1992	Norway	CSS	>60	M	n.a.	500
56	Bengtsson et al. [67]	1991	Sweden	CSS	Mean = 74	M	499	364 (72.9)

Abbreviation: AAA, asymptomatic abdominal aneurysm; CSS, Cross-sectional study; RCT, Randomized controlled trial; PCS, Prospective cohort study; W, Women; M, Men; 95% CI, 95% confidence interval; n.a., not applicable; n.r., no data or no data in appropriate format reported.

### Subgroup analysis

The prevalence of AAA ranged from 1.0% to 14.2% in men and from 0.2% to 6.4% in women. The pooled prevalence of AAA was 4.8% (4.3%, 5.3%). Pooled prevalence of all subgroups, according to geographical areas, gender, age and aneurysm diameter are presented in Table 2. The pooled prevalence of America, Europe, Australia and Asia were found to be 2.2% (2.2%, 2.2%), 2.5%(2.4%, 2.5%), 6.7% (6.5%, 7.0%) and 0.5%(0.3%, 0.7%), respectively. Male and female subgroups were 6.0% (5.3%, 6.7%) and 1.6% (1.2%, 1.9%), respectively. Prevalence in 55–64 years, 65–74 years, 75–84 years and ≥85 years were1.3% (1.2%, 1.5%), 2.8% (2.7%, 2.9%), 1.2% (1.1%, 1.3%) and 0.6% (0.4%, 0.7%), respectively. Pooled prevalence of aneurysm diameters in 30–39mm, 40–49 mm and ≥50 mm were 3.3% (2.8%, 3.9%), 0.7% (0.4%, 1.0%) and 0.4% (0.3%, 0.5%), respectively. Results showed that the pooled prevalence in Australia was higher than America and Europe. The pooled prevalence in western countries was all higher than the Asia. The prevalence of AAA in the male population was higher than in females. In addition, the prevalence in 65–74 years was the highest of the four age categories. The prevalence of aneurysms with diameters between 30 and 39 mm was higher than those with aortic diameters of more than 40 mm.

**Table 2:** Prevalence of abdominal aortic aneurysm in older people by different stratified factors.

Stratified factors	No. of Studies	Prevalence rate	Lower limit	Upper limit	Heterogeneity *I* ^2^ (%)	*P* from test of heterogeneity	Model
Total	56	0.048	0.043	0.053	99.4	0.000	REM
Area							
America	12	0.043	0.033	0.053	99.8	0.000	REM
Europe	37	0.051	0.044	0.059	98.9	0.000	REM
1988–1992	3	0.065	0.048	0.081	31.8	0.231	REM
1993–1995	6	0.065	0.036	0.094	98.3	0.000	REM
1996–1998	4	0.042	0.035	0.049	94.1	0.000	REM
1999–2001	9	0.053	0.034	0.073	99.1	0.000	REM
2002–2004	3	0.045	0.026	0.063	96.0	0.000	REM
2005–2007	2	0.047	0.032	0.062	66.4	0.085	REM
2008–2010	5	0.046	0.037	0.055	95.5	0.000	REM
2011–2013	5	0.028	0.014	0.043	99.3	0.000	REM
Australia	4	0.067	0.065	0.070	96.5	0.000	REM
Asia	3	0.005	0.003	0.007	94.6	0.000	REM
Gender							
Male	49	0.060	0.053	0.067	99.3	0.000	REM
Female	23	0.016	0.012	0.019	95.8	0.000	REM
Age (y)							
55–64	3	0.013	0.012	0.015	89.5	0.000	REM
65–74	9	0.028	0.027	0.029	97.7	0.000	REM
75–84	7	0.012	0.011	0.013	99.0	0.000	REM
≥85	2	0.006	0.004	0.007	83.2	0.000	REM
Aneurysm diameters (mm)							
30–39	11	0.033	0.028	0.039	98.3	0.000	REM
40–49	5	0.007	0.004	0.010	97.3	0.000	REM
≥50	9	0.004	0.003	0.005	90.3	0.000	REM

Abbreviation: AAA, asymptomatic abdominal aneurysm; No., number; REM, random effects model.

### Analysis of heterogeneity and publication bias

**Figure 3:**
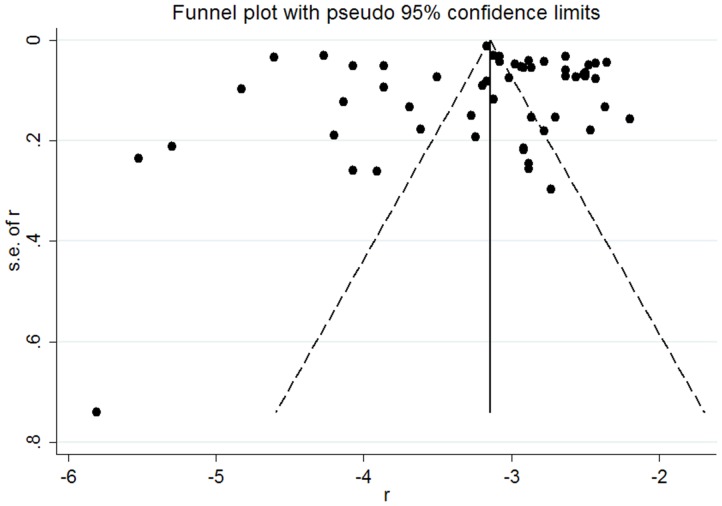
Funnel plot assessing publication bias in the prevalence of AAA from 56 published studies.

We noted significant heterogeneity within studies and subgroups (P = 0.000, I^2^ = (83.2–99.8)). The visual examination of the funnel plots (Figure 3) and Egger's test did not reveal evidence of publication bias (P = 0.863).

### Trends

**Figure 4:**
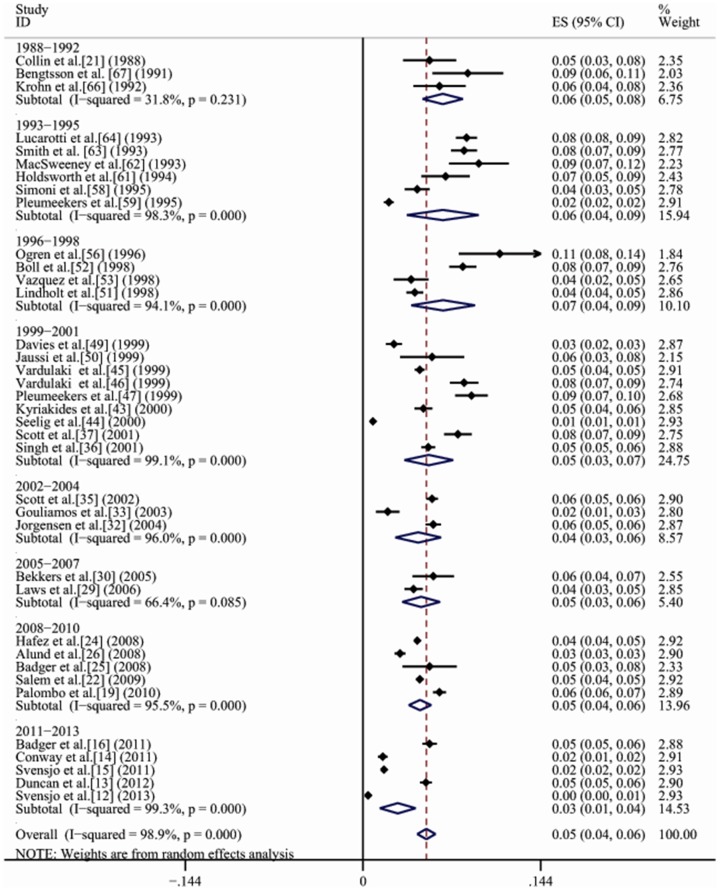
A forest plot of prevalence of AAA in Europe from 1988 to 2013.

Studies evaluating secular trends in the prevalence of AAA were available only for Europe due to paper quantitative restrictions. Time trend analyses based on years of fieldwork showed that the prevalence of AAA for general population in Europe gradually decreased from 6.5% (95% CI, 4.8%–8.1%) in 1988–1992 to 2.8% (95% CI, 1.4%–4.3%) in 2011–2013 (Figure 4).

### Risk factors

Only 12 studies (nine among Europe, one among America, one among Australia and one among Asia) reported on risk factors for AAA. Table 3 gives combined odds ratios calculated by random or fixed-effects models and probabilities from tests of heterogeneity. Hypertension, smoking, coronary artery disease, dyslipidemia, respiratory disease, cerebrovascular disease, claudication and renal insufficiency were risk factors for AAA in Europe; Smoking and coronary artery disease were risk factors for AAA in America; Smoking, diabetes mellitus, coronary artery disease, dyslipidemia and respiratory disease were risk factors for AAA in Australia; Hypertension and smoking were risk factors for AAA in Asia.

**Table 3:** Combined odds ratios for the presence of abdominal aortic aneurysm from meta-analysis.

Risk factor (yes vs. no)	Number of studies (bibliography number)	Combined odds ratio (95% CI)	*p* from test of heterogeneity
Hypertension			
Total	12 (12,15,17,19,20,32,38,53,58,59,63,67)	1.26 (1.15, 1.39)	0.000
Europe	9 (12,15,19,32,53,58,59,63,67)	1.31 (1.17, 1.47)	0.000
America	1 (38)	1.08 (0.89, 1.33)	-
Australia	1 (17)	1.27 (0.93, 1.74)	-
Asia	1 (20)	2.35 (1.02, 5.43)	-
Smoking (previous or current)			
Total	11 (12,15,17,19,20,32,38,53,58,59,63)	2.07 (1.87, 2.28)	0.000
Europe	8 (12,15,19,32,53,58,59,63)	1.93 (1.72, 2.16)	0.000
America	1 (38)	1.79 (1.27, 2.28)	-
Australia	1 (17)	4.03 (2.75, 5.90)	-
Asia	1 (20)	3.29 (1.43, 7.61)	-
Diabetes mellitus			
Total	10 (12,15,17,19,20,32,38,53,58,59,63)	1.04 (0.90, 1.19)	0.007
Europe	7 (12,15,19,32,53,58,59,63)	0.97 (0.81, 1.17)	0.073
America	1 (38)	0.87 (0.63, 1.21)	-
Australia	1 (17)	1.65 (1.21, 2.23)	-
Asia	1 (20)	0.54 (0.18, 1.61)	-
Coronary artery disease			
Total	10 (12,15,17,19,32,38,58,59,63,67)	1.82 (1.65, 2.00)	0.000
Europe	8 (12,15,19,32,58,59,63,67)	1.55 (1.37, 1.76)	0.000
America	1 (38)	1.91 (1.55, 2.35)	-
Australia	1 (17)	3.15 (2.44, 4.07)	-
Dyslipidemia			
Total	7 (12,15,17,19,20,53,58)	1.36(1.19, 1.54)	0.000
Europe	7 (12,15,19,53,58)	1.31(1.13, 1.50)	0.000
Australia	1(17)	1.78(1.30, 2.43)	-
Asia	1(20)	0.25(0.06, 1.07)	-
Respiratory disease			
Total	6 (12,15,17,19,58,63)	1.36 (1.19, 1.55)	0.000
Europe	5(12,15,19,58,63)	1.35 (1.16, 1.58)	0.000
Australia	1(17)	1.36 (1.04, 1.77)	-
Cerebrovascular disease			
Total	5 (12,15,32,58,59)	1.28 (0.93, 1.77)	0.070
Europe	5 (12,15,32,58,59)	1.28 (0.93, 1.77)	0.070
Claudication			
Total	3 (12,15,59)	3.00 (1.74, 5.19)	0.330
Europe	3 (12,15,59)	3.00 (1.74, 5.19)	0.330
Renal insufficiency			
Total	3 (12,15,19)	1.20(0.95, 1.51)	0.110
Europe	3 (12,15,19)	1.20(0.95, 1.51)	0.110

## Discussion

The aim of this meta-analysis was to estimate prevalence rates of AAA in general population. To our knowledge, this is the first meta-analysis examining the prevalence of AAA in general population. 56 epidemiological studies were selected. Our analysis suggested that approximately 4.8% of the general population has AAA (6.0% for males and 1.6% for females). Results show that the pooled prevalence in Australia is higher than America and Europe. The pooled prevalence in western countries is higher than the Asia. The present meta-analysis indicated that the prevalence of AAA has decreased in Europe from 1988 to 2013. The prevalence of AAA in the male population is higher than in females. In addition, the prevalence in 65–74 years is the highest of the four age categories. The prevalence of aneurysms with diameters between 30 and 39 mm is higher than those with aortic diameters of more than 40 mm. The population prevalence of AAA varied widely which is not surprising considering the differences between studies in terms of their definition of AAA, area difference, age and gender distribution of study populations.

Necropsy reports provided the first information on AAA epidemiology. From Malmö, Sweden, a prevalence of 4.7% in men and 1.2% in women who were 65 to 74 years was reported [68] (10,413 necropsies with a 70% necropsy ate). Estimates of the prevalence of AAA can also be obtained from screening surveys. The reported prevalence of screening-detected AAA varies depending on the areas, gender, age, aortic diameters, and the criteria used to define an AAA.

Our study shows that prevalence of AAA differs in areas. The prevalence in Australia is higher than America and Europe, and the prevalence in western countries is higher than the Asia. Presently, the world is embracing various large epidemiological studies which assess the current situation of AAA. A screening study done in the USA found 31 AAA in 2005 residents who aged over 65 years.[23] Another UK study screened 4216 residents and found only 69 patients with AAA (1.6%).[14] In the Australia, with the population of about 3620, 262 AAA was found in the community (7.2%). [17] However, studies from Japan and Korea reported a relatively low prevalence of AAA in Asians. [20], [42] Accurate data on AAA in the Asian population are very limited. A Hong Kong study screened total population and found only 0.14% patients with AAA. [69] However, there are few epidemiological studies in mainland China. In mainland China, research on causes of AAA has just started. Researches about causes focus on basic studies including animal and vitro tests. Population-based epidemiological studies are small in scale and sample size, however. Most researches are hospital-based single center and small studies. It needs continuousconclusion and perfectibility.

Prevalence of AAA showed a greater gender gap. Our meta-analysis confirmed this result. The pooled prevalence of AAA in males is higher than in females, 6.0% and 1.6%, respectively. AAA primarily affects men, who have a 5-fold greater prevalence of AAA compared with women in studies using ultrasound screening. [36], [57], [59] The prevalence of AAA has been reported as 1.3–8.9% in men and 1.0–2.2% in women in Western countries. [1], [70], [71] But it's important to note that the mortality rate associated with ruptured AAA among women is increasing, and that the rate of rupture is higher in women than in men. [72], [73] Female patients with AAA were only one 5th that of male patients, but one patient with AAA in three ruptured was female. [58]

Postmortem studies have suggested that 95% of deaths from ruptured AAA occur at or above the age of 65 years. It has, therefore, been recommended to focus screening at age 65 years to maximize the potential number of life years gained. In Bekkers et al. group [30]AAA started to occur at age 55 years with two patients already having significantly dilated abdominal aortas before the age of 60 years, with diameters of 55 and 68 mm, respectively. AAA was not found before the age of 50 years in both sexes, but the prevalence increased with age for both men and women. A one-time screening of men aged 60 to 65 years has also been shown to be cost-effective. [74], [75] After the age of 70 years AAA increased significantly only in men.

The majority of abdominal aorta diameters were between 30 and 40 mm. The rate of growth of abdominal aneurysms is relatively unpredictable with wide interindividual variability but seems to be increased in larger aneurysms. The mean expansion rate of AAA has been estimated to vary between 0.28 and 0.38 cm/year. [76], [77] All diameters were under 4 cm with low risk of rupture. [8] A second screening in patients with aortic diameters less than 30 mm has been shown to be of little practical value and is, therefore, not recommended. However, the following recommendations for subsequent surveillance have been made: patients with AAA between 3 and 4 cm should have an ultrasound after 1 year, between 4 and 4.5 cm after 6 months, and greater then 4.5 cm should be referred to a vascular surgeon.

Prevalence of AAA has been increasing for the past two decades, which possibly correlates to increased average life span and development of diagnostic tools and screening programs.[78] AAA screening in general population reported the prevalence of AAA from 1% to 7% of the general Western population [30], [31], [54] and 5% of men over 65 years of age. [79] A ruptured AAA can be fatal; therefore, a screening program is recommended for populations at increased risk. Currently, the U.S. Preventive Services Task Force recommends an ultrasound for AAA screening in men aged 65–75 years who have ever smoked. [80] This program has achieved reduced mortality in men aged 65–74 years. [79]

The population prevalence of AAA varied from region to region which is not surprising considering the differences between studies in terms of their definition of AAA, the age and sex distribution of study populations and the prevalence of risk factors. The results showed that hypertension, smoking, coronary artery disease, dyslipidemia, respiratory disease, cerebrovascular disease, claudication and renal insufficiency were risk factors for AAA in Europe; Smoking and coronary artery disease were risk factors for AAA in America; Smoking, diabetes mellitus, coronary artery disease, dyslipidemia and respiratory disease were risk factors for AAA in Australia; Hypertension and smoking were risk factors for AAA in Asia. Aortic aneurysms are a complex genetic disorder with environmental risk factors. The exact pathogenesis of abdominal aortic aneurysm has not been completely unraveled. It is clear, however, that it involves a series of known and unknown environmental factors acting over time. It is not known exactly which genetic risk factors make a person prone to aortic wall dilatation. Familial aggregation of AAA suggests that there are candidate genes that contribute to the development of AAA. The magnitude of the increased risk in first degree relativ**es** suggests a genetic component, although the influence of a common lifestyle cannot be excluded. A recent molecular genetic study in an Irish population found no significant gene–disease associations.

A few limitations of this meta-analysis must be considered. First, the literature search was limited to articles published in English or Chinese. Nonetheless, no evidence of publication bias was found. Second, some characteristics of the subjects, such as ethnicity, which might exert an important influence on the prevalence of AAA, were not included in the meta-analysis. Finally, there is no general agreement on how to define an AAA. Moher et al. [81] demonstrated how various definitions strongly influence the reported prevalence of AAA, a finding confirmed by means of this study. Steinberg et al. [82] established normal standards for abdominal aortic diameters. They concluded that a diameter in excess of 30 mm was well above the average for both sexes and was considered to be the dividing line between ectasia and aneurysms. [83] This was the basis for the most accepted definition, described by McGregor et al. [84] in 1975, which defined an AAA as a maximum intracranial aortic diameter of 30 mm or more. Because it is widely used, there are several studies with which to compare when this definition is chosen, and there is no need to define the individuals according to age, sex, and body surface area (BSA) to calculate the normal aortic diameter.

Rupture of an AAA is fatal, and mortality is more than 50% before arrival at a hospital. Even if a patient survives the trip to the operating room, operation-related mortality has been described up to 70%. [85], [86] A routine screening for AAA during clinical transthoracic echocardiography (TTE) provides a low yield due to a low prevalence (0.5%) of AAA in general population. However, the detection of life-threatening but asymptomatic AAA may save lives. Therefore, a routine examination of the abdominal aorta during TTE, which involves little additional time, would appear to be an effective and efficient prevention strategy, especially in men over 60 years of age. When the cost is covered by governments, priorities have to be decided on the basis of the total budget and the need for screening of other diseases. On an individual basis, however, we must state that each person has the right to know what kind of disease may possibly affect him, and to decide whether to be screened or not, at his own expense.

## Conclusion

AAA is common in general population. The prevalence of AAA is higher in Australia than America and Europe. The pooled prevalence in western countries is higher than the Asia. A higher prevalence of AAA is also found in 65–74 years and among males. The prevalence of aneurysms with diameters between 30 and 39 mm is higher than those with aortic diameters of more than 40 mm.

## Supporting Information

Checklist S1 **PRISMA Checklist.** Doi:10.1371/journal.pone.0081260.s001
(DOC)
